# Left anomalous coronary artery originating from the opposite sinus causes acute myocardial infarction with syncope in a young woman: A case report

**DOI:** 10.1097/MD.0000000000039850

**Published:** 2024-09-27

**Authors:** Cheng Zhang, Dan Shi

**Affiliations:** a Department of Cardiology, Jilin University Bethune Third Clinical Hospital, Changchun, Jilin Province, P. R. China; b Department of Radiotherapy, Jilin University Bethune Third Clinical Hospital, Changchun, Jilin Province, P. R. China.

**Keywords:** acute myocardial infarction, L-ACAOS, syncope

## Abstract

**Rationale:**

The left anomalous coronary artery originating from the opposite sinus represents a distinct subtype of anomalous coronary arteries originating from the opposite sinus. A comprehensive overview encompassing clinical characteristics, diagnostic approaches, and treatment modalities for this condition is presented herein.

**Patient concerns:**

The patient, a 17-year-old female, was admitted to the hospital due to chest pain and syncope following multiple exercises.

**Diagnoses:**

After conducting an electrocardiogram, myocardial injury markers, and echocardiography, she was diagnosed with acute myocardial infarction complicated by syncope. Further examinations including coronary angiography, coronary computed tomography angiography, and cardiac magnetic resonance imaging revealed an anomalous origin of the coronary artery with the left coronary artery (LCA) arising from the right sinus and exhibited intramural course.

**Interventions:**

The coronary “unroofing” technique was admitted according to her characteristics. The patient achieved successful recovery after surgery with no recurrence of chest pain or syncope during 1 year of follow-up.

**Outcomes:**

Anomalous origin of the LCA is a rare congenital anatomical anomaly. Surgical intervention represents the primary approach for subsequent management of symptomatic anomalous origin of the coronary artery. Importantly, individuals with anomalous origin of the LCA from the right coronary sinus are at potential risk of sudden cardiac death.

**Lessons:**

Therefore, enhancing diagnostic precision and actively pursuing surgical treatment in confirmed diagnoses can effectively mitigate myocardial ischemia and prevent instances of sudden cardiac death among adolescents and athletes.

## 1. Introduction

Left anomalous coronary artery originating from the opposite sinus (L-ACAOS) represents a specific subtype of anomalous coronary arteries originating from the opposite sinus (ACAOS), which is considered a congenital coronary artery disease responsible for the ectopic positioning of the left coronary artery (LCA) within the sinus. These structural abnormalities are implicated in syncope and sudden cardiac death (SCD) following physical exertion among numerous young individuals. Notably, there has been 1 reported case of syncope attributed to anomalous origins of the LCA arising from the right coronary sinus.^[1]^ A comprehensive overview encompassing clinical characteristics, diagnosis, and treatment history pertaining to this condition is presented herein.

## 2. Case report

**Figure 1.**
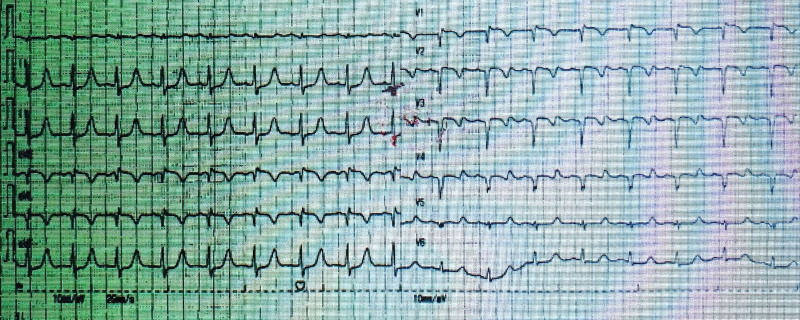
ECG: ST-segment depression in leads II, III, and aVF, ST-segment elevation in lead avR and lead V1–V3, Q waves were showed in lead V1–V4.

**Figure 2.**
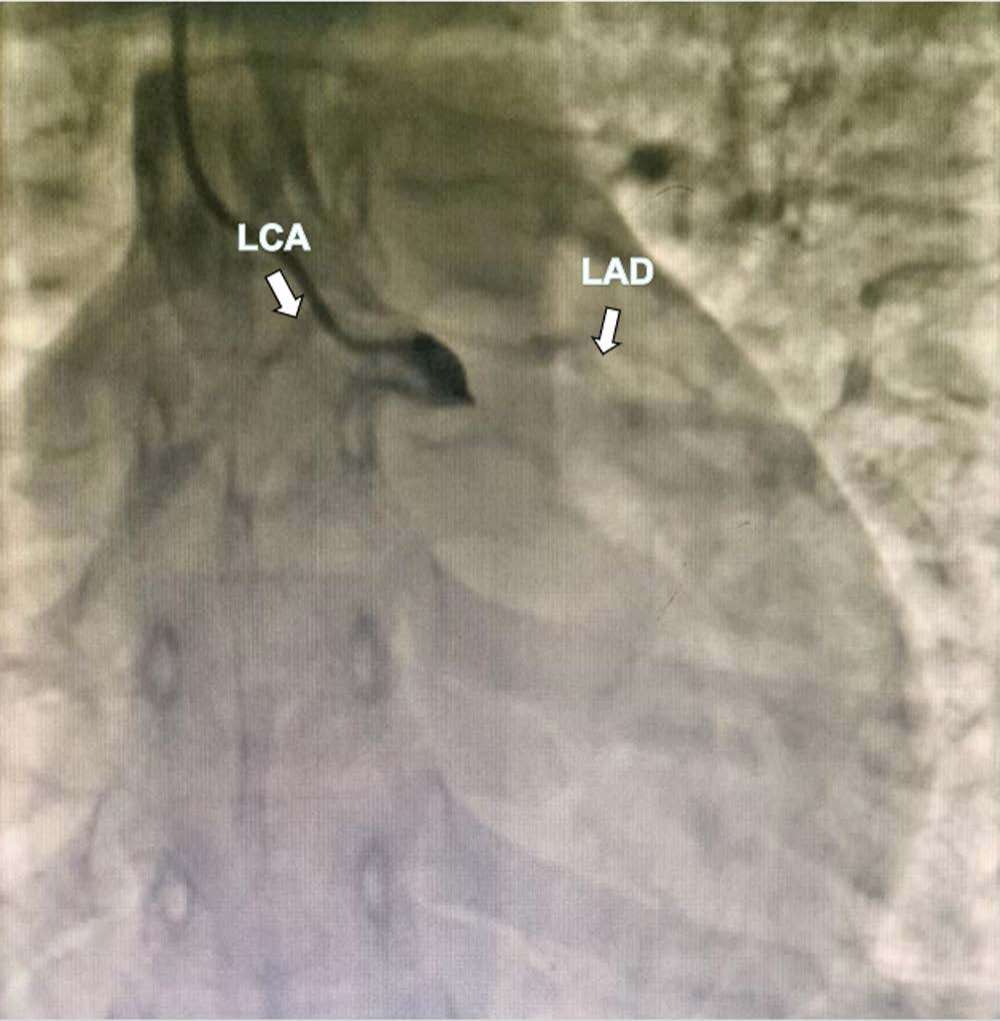
Left coronary angiography. Failure to find the left coronary opening, nonselective angiography was performed to suspect 90% stenosis of the left main coronary artery.

**Figure 3.**
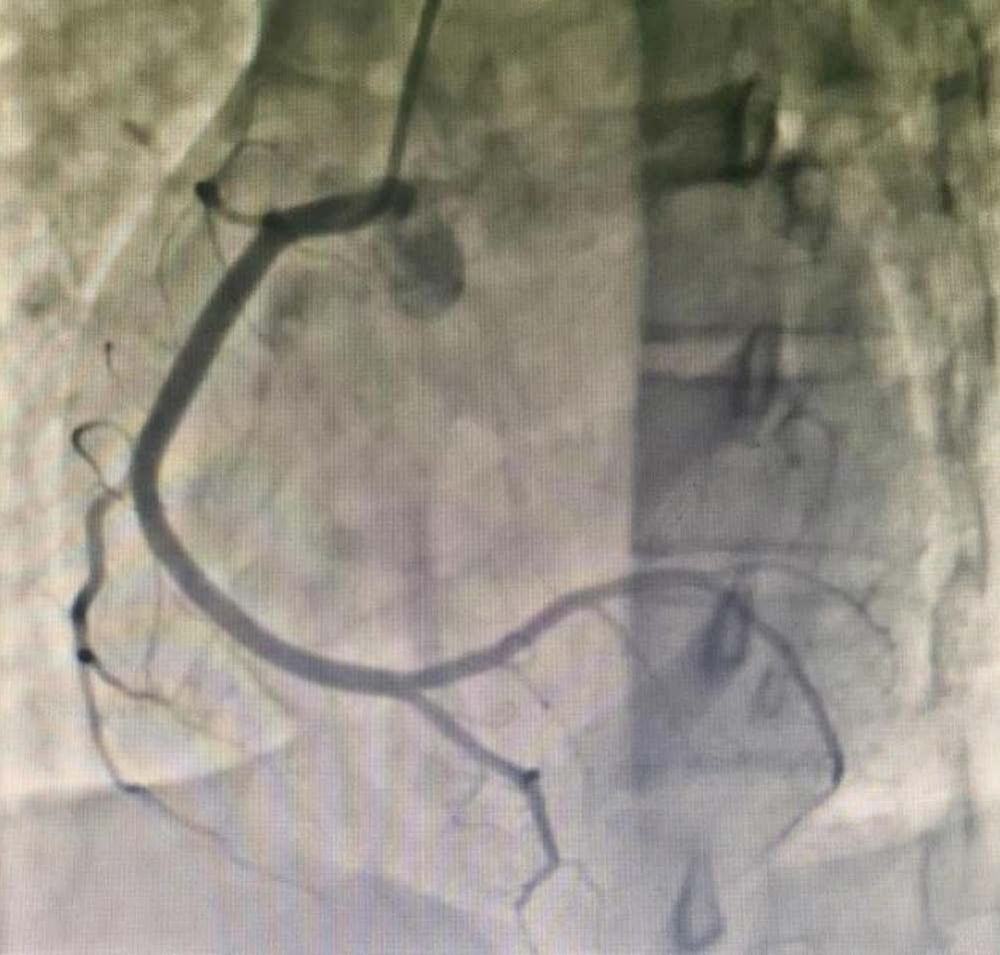
Angiography of the right coronary artery was normal.

**Figure 4.**
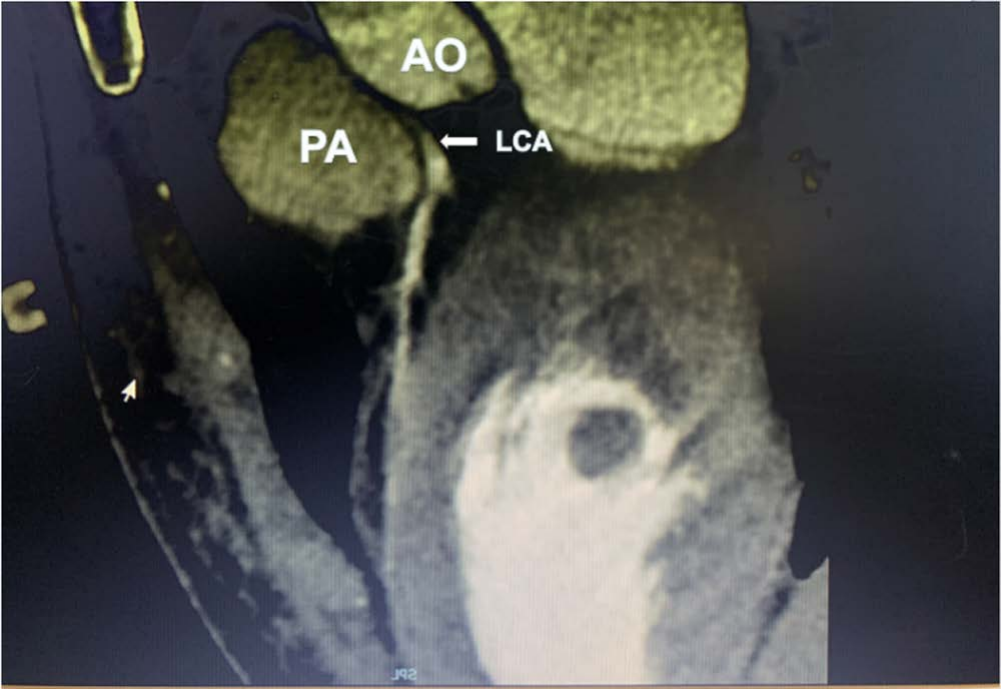
CCTA: LCA is located between the Ao and the Pa.

**Figure 5.**
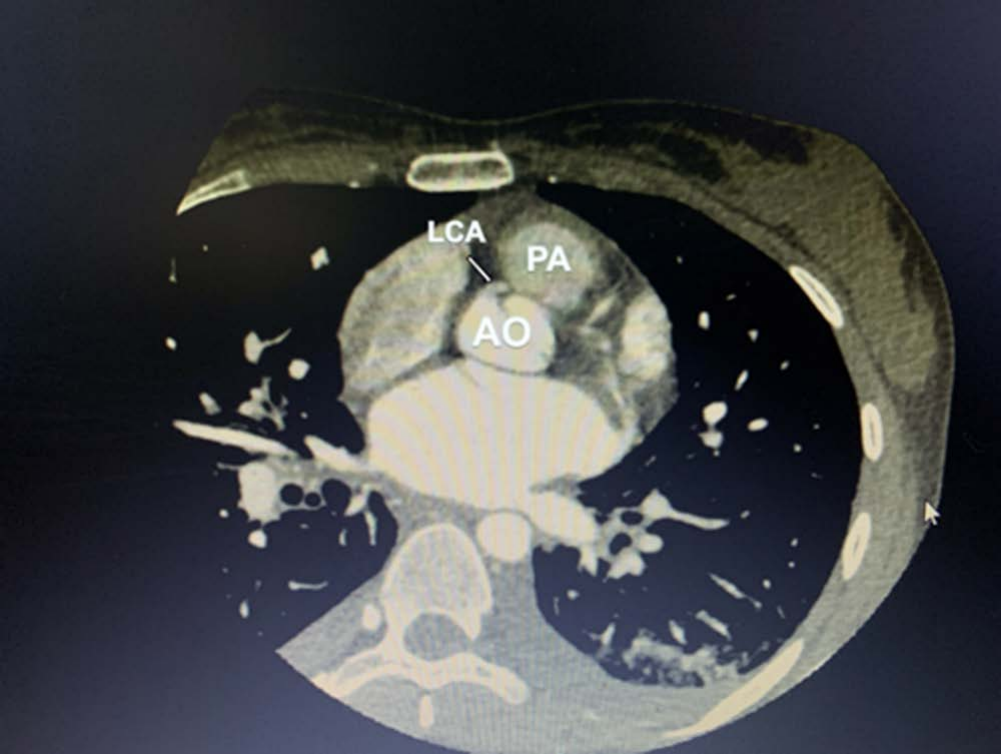
Coronary CTA: LCA is located between the Ao and the Pa according to axial section, with originating from the right sinus and exhibiting intramural course.

**Figure 6.**
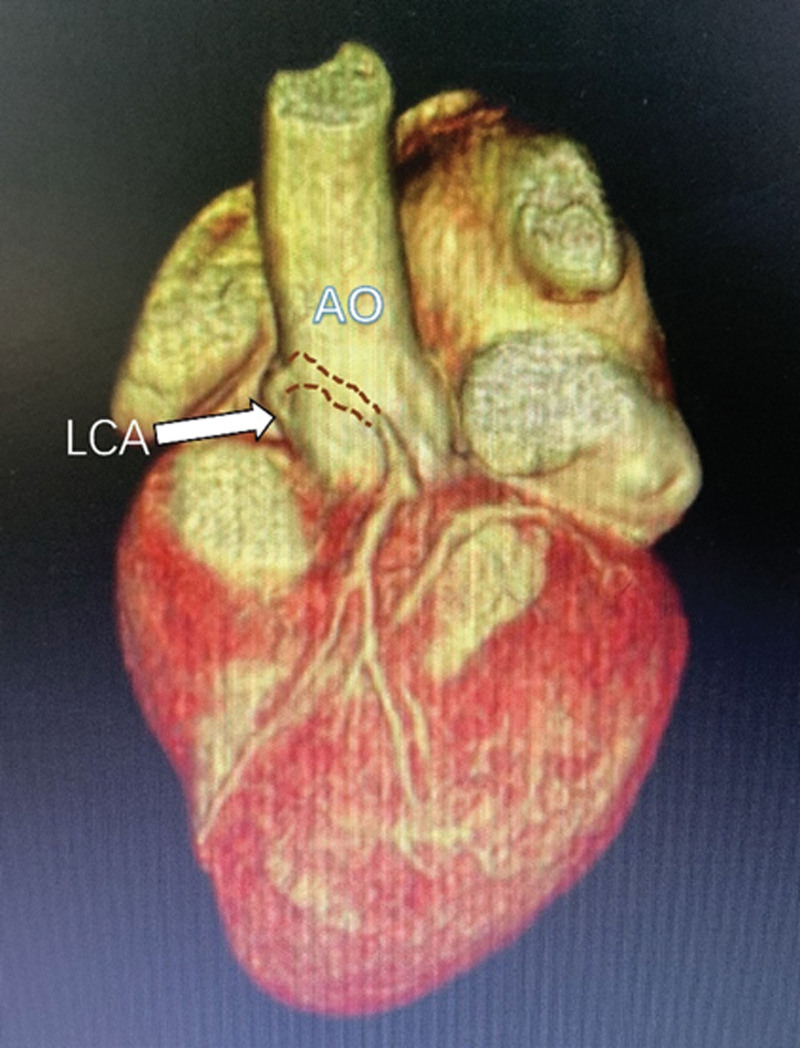
Coronary CTA: LCA is originated from the opposite sinus, and exhibited intramural course.

A 17-year-old female, was admitted to the hospital due to chest pain and syncope following multiple exercise sessions. In the beginning, at the age of 7, she experienced chest pain accompanied by syncope after exercising; however, no abnormalities were detected in the electrocardiogram (ECG) or markers of myocardial injury, and no further treatment was administered. Four years ago (at 13 years old), she again experienced chest pain after running, which was followed by syncope. An examination at a local hospital revealed elevated levels of CK and CK-MB while maintaining normal results in the ECG and echocardiography (ECHO). Ten days prior to admission, the patient experienced recurrent chest pain following physical exertion along with symptoms of dizziness, nausea, and subsequent syncope. ECG findings showed ST-segment depression in leads II, III, and aVF, ST-segment elevation in lead aVR and lead V1–V3, Q waves were showed in lead V1–V4 (Fig. 1). ECHO revealed impaired left ventricular function (LVEF) with an ejection fraction (EF) of 32%. Regional wall motion abnormalities were found in basal and mid segments of anterior septum. Additionally observed were elevated markers of myocardial injury: cardiac troponin I (cTnI) at 14044.6 pg/mL; myoglobin at 15.3 ng/mL; CK at 208 U/L; and BNP at 953.0 pg/mL. Coronary angiography (CAG) was performed: the conventional angiography catheter failed to visualize the left coronary ostium, while no abnormalities were observed in the right coronary artery. Nonselective angiography indicated a suspected 90% stenosis of the left main coronary artery (Figs. 2 and 3). Cardiac magnetic resonance (CMR) imaging revealed impaired LVEF (EF: 29%), subendocardial (or transmural) ischemia of the septum and of the antero-lateral wall of the left ventricle, with a concomitant diffuse edema. Coronary computed tomography angiography (CCTA) revealed an anomalous origin of the coronary artery, with the LCA arising from the right sinus (Figs. 4–6). Intraoperatively, it was observed that the LCA originated from the right sinus and exhibited intramural course. The coronary “unroofing” technique was admitted according to her characteristics. Subsequently, the patient experienced a favorable recovery without any episodes of chest pain or syncope and administered β-receptor blockade (6.25 mg metoprolol, 3 times daily). Six-month follow-up echocardiogram revealed successful repair with relocation of L-ACAOS, resulting in a preserved LVEF of 50% along with mild mitral regurgitation and mild tricuspid regurgitation. Summary of events were showed in Table 1.

**Table 1:** Summary of events.

Age	Syndromes	Events
7	Chest pain; syncope	No abnormal.
13	Chest pain; syncope	Markers of myocardial injury were abnormal; ECG and ECHO were normal.
17	Chest pain; syncope; dizziness; nausea	Markers of myocardial injury were abnormal; CAG, CTA, CMR, ECG, and ECHO were abnormal.

## 3. Discussion

The ectopic sinus of the LCA is a rare congenital anatomical anomaly that has been infrequently reported in medical literature. Available studies suggest an incidence ranging from 0.6% to 5.64%.^[2,3]^ Although relatively infrequent, coronary artery abnormalities can account for up to 15% of cases involving syncope and SCD, rendering its second most prevalent cause of SCD in young athletes following hypertrophic cardiomyopathy.^[4]^ Symptoms associated with myocardial ischemia are frequently observed during or following exercise and may encompass angina pectoris, dyspnea, ventricular arrhythmias, and instances of SCD. It is noteworthy that SCD can occasionally manifest as the initial symptom in certain patients. The primary anatomical factors contributing to these symptoms include the positioning of the LCA between the Ao and pulmonary artery (Pa), as well as its course within the aortic wall. Specifically, compression on the intramural LCA occurs due to both the Ao and Pa trunk, which is further exacerbated by external factors such as physical activity. This compression leads to increased tension within the aortic wall and subsequent stenosis (narrowing) of the LCA. Ultimately, this can result in myocardial infarction (heart attack) and SCD, particularly among young athletes.

Presently, it is recommended for patients suspected of having an anomalous origin of the LCA to undergo ECHO, CCTA, and CMR. These noninvasive techniques have exhibited substantial effectiveness in detecting a considerable number of cases.^[5]^ The guidelines from the American College of Cardiology (ACC) and the American Heart Association (AHA) underscore the criticality of early surgical revascularization in managing congenital heart disease in adults, particularly for patients with L-ACAOS, as well as abnormal aortic and Pa.^[6]^ However, the frequent misdiagnosis as fulminant myocarditis or cardiomyopathy underscores the critical importance of rapid and accurate diagnosis in determining prognosis and preventing SCD associated with ACAO. Therefore, employing multiple imaging strategies is considered a reasonable approach for effectively screening patients’ coronary anatomy and achieving precise diagnostic outcomes.^[7]^ The CCTA technique is highly accurate in determining both the precise location and intricate details of internal segment morphology, while also providing an assessment of the angle between the opening and proximal segment.^[8,9]^ The CMR provides a comprehensive evaluation, incorporating functional imaging capabilities that are particularly valuable for assessing the extent and severity of myocardial infarction.^[10]^ Additionally, although the invasive nature of CAG limits its routine application, it can still be used as a supplementary method to determine complex anatomical variations. This is particularly relevant considering that noninvasive methods such as CCTA or CMR effectively fulfill most imaging objectives.

ECG serves as a valuable diagnostic tool for patients presenting with anomalous origin of the LCA. Specifically, ST-segment elevation observed in lead aVR is indicative of LCA disease.^[11]^ However, the presence of ST-segment elevation in lead aVR in a younger patient without traditional risk factors for coronary artery disease, as described in our report, should raise suspicion of potential coronary abnormalities. Further studies and case reports are necessary to validate this phenomenon.

Regarding surgical techniques, there are specific recommendations for each technique, particularly highlighting their characteristics.^[12]^ The coronary “unroofing” technique is widely recognized as the most commonly performed surgical procedure for LCA originated from the right sinus and exhibited intramural course.^[13]^ In a cohort study involving 45 North American centers from the Congenital Heart Surgeons’ Society (CHSS), isolated unroofing was found to be the predominant repair strategy (87%), followed by unroofing with commissural manipulation (25%), patch ostioplasty (6%), reimplantation (6%), Pa translocation (6%), and other strategies.^[14]^

The patient, a young woman without known risk factors such as coronary heart disease, underwent comprehensive examinations to exclude diseases commonly associated with myocardial infarction in adolescents, including Kawasaki disease and Takayasu arteritis. In conjunction with clinical manifestations, ECG findings, troponin levels, CCTA results, and CAG findings were utilized to confirm the presence of ACAO along with severe compression of the left main trunk. CMR revealed multiple focal necrosis within the supply area of the LCA further supporting this anomaly. Consequently, myocardial ischemia occurred following activity resulting in myocardial ischemia. Given its crucial role in blood supply distribution, any compromise to the left main coronary artery can lead to fatal arrhythmias, sudden death, acute pulmonary edema, cardiogenic shock as well as subsequent heart failure and poor prognosis post-ischemia.

This patient has experienced syncope on 2 occasions during physical exertion, and cardiogenic disease is a well-established etiology of syncopal episodes. Additionally, Rigatelli et al^[15]^ have reported cases of syncope associated with ACAO, indicating that patients presenting with unexplained syncope should consider investigating the possibility of an anomalous LCA origin. The presence of ST-segment elevation in lead aVR on the ECG strongly suggests left main or three-vessel coronary artery disease. Timely revascularization when indicated, prompt utilization of advanced imaging techniques for coronary assessment, and early recognition of clinical signs and ECG findings indicative of myocardial ischemia are crucial in reducing mortality rates among patients with ACAO.^[16]^ Surgical intervention is the primary therapeutic approach for managing symptomatic abnormal origin of the coronary arteries.^[17,18]^ In this specific case, successful unroofing surgery effectively alleviated constriction in both the Ao and Pa, while restoring normal anatomical structure to the affected coronaries. Individuals with aberrant origin of the LCA from the right sinus face an elevated risk of SCD; therefore, it is crucial to enhance our understanding of this anomaly, particularly among adolescents and athletes. To mitigate myocardial ischemia and prevent SCD, there should be an increase in diagnostic rates alongside active provision of surgical therapy for confirmed cases.

## 4. Conclusion

ACAO is a rare congenital anatomical anomaly. Surgical intervention represents the primary approach for subsequent management of symptomatic patients. Importantly, individuals with L-ACAOS are at potential risk of SCD. Therefore, enhancing diagnostic rates and actively pursuing surgical treatment in confirmed diagnoses can effectively mitigate myocardial ischemia and prevent instances of SCD among adolescents and athletes.

## Author contributions

**Data curation:** Dan Shi.

**Resources:** Cheng Zhang.

**Writing – original draft:** Cheng Zhang.

**Writing – review & editing:** Dan Shi.
